# Association between the dietary inflammatory index and depressive symptoms in adults with metabolic syndrome: a cross-sectional study from the NHANES database

**DOI:** 10.3389/fnut.2025.1518551

**Published:** 2025-01-27

**Authors:** Jinshun You, Shujie Xia

**Affiliations:** ^1^Fujian University of Traditional Chinese Medicine, Fuzhou, China; ^2^The Second People’s Hospital Affiliated to Fujian University of Traditional Chinese Medicine, Fuzhou, China

**Keywords:** diet, depression, inflammation, adult population, database

## Abstract

**Objective:**

The comorbidity of metabolic syndrome (MS) and depressive symptoms has emerged as a growing public health concern, contributing to a substantial global economic burden. The pathogenesis of this comorbidity is thought to be closely linked to inflammation. However, research examining the impact of the Dietary Inflammatory Index (DII) on depressive symptoms in adults with MS remains limited. This study aims to investigate the association between the DII and depressive symptoms in adults with MS, utilizing data from the National Health and Nutrition Examination Survey (NHANES).

**Methods:**

This cross-sectional study included 7,553 participants aged 20 and older MS from six cycles of the NHANES (2007–2018). Depressive symptoms were assessed using the Patient Health Questionnaire scores, and dietary information was gathered to calculate the Dietary Inflammatory Index (DII). The association between DII scores and depressive symptoms in individuals with MS was evaluated through multivariable logistic regression analysis, adjusting for relevant covariates. Subgroup analyses and restricted cubic splines (RCS) were performed to explore this relationship further.

**Results:**

Among the participants, 907 individuals (12.0%) were identified as having depressive symptoms. After adjusting for all covariates, a positive correlation was observed (OR = 1.09, 95% CI: 1.04–1.14). After adjusting for all covariates, a positive association was observed between DII scores and depressive symptoms (OR = 1.09, 95% CI: 1.04–1.14). Individuals in the highest tertile of DII scores had significantly higher odds of depressive symptoms compared to those in the lowest tertile (OR = 1.36, 95% CI: 1.13–1.65). Subgroup analyses revealed that men were more likely to experience depressive symptoms among adults with MS. The RCS model revealed a nonlinear positive association between DII scores and depressive symptoms in these participants.

**Conclusion:**

Our study indicates that the DII is positively correlated with an increased likelihood of depressive symptoms among adults with MS in the United States. These findings align with existing research and necessitate further validation through prospective cohort studies.

## 1 Introduction

Metabolic syndrome (MS) is a complex metabolic disorder characterized by a cluster of interrelated risk factors, including abdominal obesity, hypertension, hyperglycemia, and dyslipidemia. Driven largely by adverse lifestyle factors, the global prevalence of MS continues to rise, positioning it as a significant risk factor for the development of cardiovascular diseases and diabetes (1). Depressive states are primarily characterized by persistent low mood, sadness, or distress. When these symptoms evolve into a chronic mood disorder that substantially interferes with daily functioning, they are clinically recognized as depressive symptoms. The World Health Organization predicts that by 2030, depressive symptoms will be the leading cause of disease burden in high-income countries (2). Both MS and depressive symptoms present substantial public health challenges. Emerging evidence increasingly suggests a significant association between MS and depressive symptoms. A long-term study spanning 21 years has shown that the incidence of hyperglycemia and dyslipidemia is closely linked to the development of depressive and anxiety disorders (3). Koponen et al. (4). observed that the incidence of developing depressive symptoms after a diagnosis of MS is twice as high as in individuals without MS. Research has shown that insulin resistance, elevated cortisol levels, and immune-inflammatory activation associated with MS are closely linked to the onset of depressive symptoms. Moreover, people with comorbid MS and depressive symptoms tend to experience more complex symptomatology, lower recovery rates, and frequent recurrence of episodes (5).

Compared to other indices reflecting dietary quality, such as the Healthy Eating Index-2015 (6), the Dietary Inflammatory Index (DII) is specifically designed to objectively integrate evidence from diverse research methods and nutritional assessments across various populations. The DII score provides a measure of the inflammatory potential of an individual’s diet, with higher scores indicating a greater pro-inflammatory effect (7). The DII pattern can promote systemic inflammation, which is closely associated with abnormal levels of inflammatory markers in the bloodstream, including C-reactive protein, tumor necrosis factor-*α*, and interleukin (IL)-1β. This relationship underscores the critical role of diet in modulating inflammation and its potential impact on various health outcomes (8).

The pathogenesis of MS primarily involves insulin resistance, impaired lipolysis, and excessive accumulation of fatty acids (1). Dietary factors play a crucial role in these processes, with diets low in saturated fats and rich in fiber, monounsaturated oils, vitamins, and minerals exhibiting protective effects against the development of MS (9). Conversely, diets high in meat consumption and fried foods, characteristic of Western dietary patterns, have been linked to the onset of MS (10). Unhealthy dietary habits can induce adverse biological effects that contribute to the initiation and progression of MS. For instance, high-fat diets disrupt energy metabolism by promoting lipid accumulation, which adversely affects adipose tissue metabolism and contributes to obesity (11). A systematic review has shown that higher DII scores are associated with key components of MS, including hypertension, hyperglycemia, abdominal obesity, and dyslipidemia (12).

Despite the incomplete understanding of the etiology of depressive symptoms, current research has shown a significant correlation between depressive symptoms and the expression of inflammatory molecules (13). Studies indicate that an imbalance between pro-oxidants and antioxidants increases the production of reactive oxygen species, leading to oxidative stress, which may be linked to the pathogenesis of depressive symptoms (14). Foods rich in various vitamins exhibit anti-inflammatory and antioxidant properties, and they can also modify gut bacteria strains, thereby regulating gut inflammation associated with depressive symptoms (13). Cross-sectional studies have shown that breastfeeding women can reduce the incidence of postpartum depressive symptoms by adjusting their dietary patterns to lower the inflammatory score of their diet (15). Thus, it is evident that an inflammatory dietary pattern can influence MS and depressive symptoms. However, it remains unclear whether such a dietary pattern may exacerbate depressive symptoms in people with MS. To address this gap, we utilized data from the National Health and Nutrition Examination Survey (NHANES) from 2007 to 2018 to investigate the relationship between the DII and the likelihood of depressive symptoms in U.S. adults with MS.

## 2 Methods

### 2.1 Data source and study population

The Centers for Disease Control and Prevention conducted a nationally representative survey called NHANES. The research ethics review committee of the National Center for Health Statistics (NCHS) approved the research procedure (2017–2018: Protocol#2018-01 Effective beginning October 26, 2017; Continuation of Protocol#2011-17 Effective through October 26, 2017. 2015–2016: Continuation of Protocol#2011-17. 2013–2014: Continuation of Protocol#2011-17. 2011–2012: Protocol#2011-17. 2009–2010: Continuation of Protocol #2005-06. 2007-2008:n Continuation of Protocol#2005-06.) The NCHS Protocol Number are available at https://www.cdc.gov/nchs/nhanes/about/erb.html. Some participants provided written consent at the time of recruitment. NHANES data can be publicly available on https://www.cdc.gov/nchs/nhanes/.

**Figure 1:**
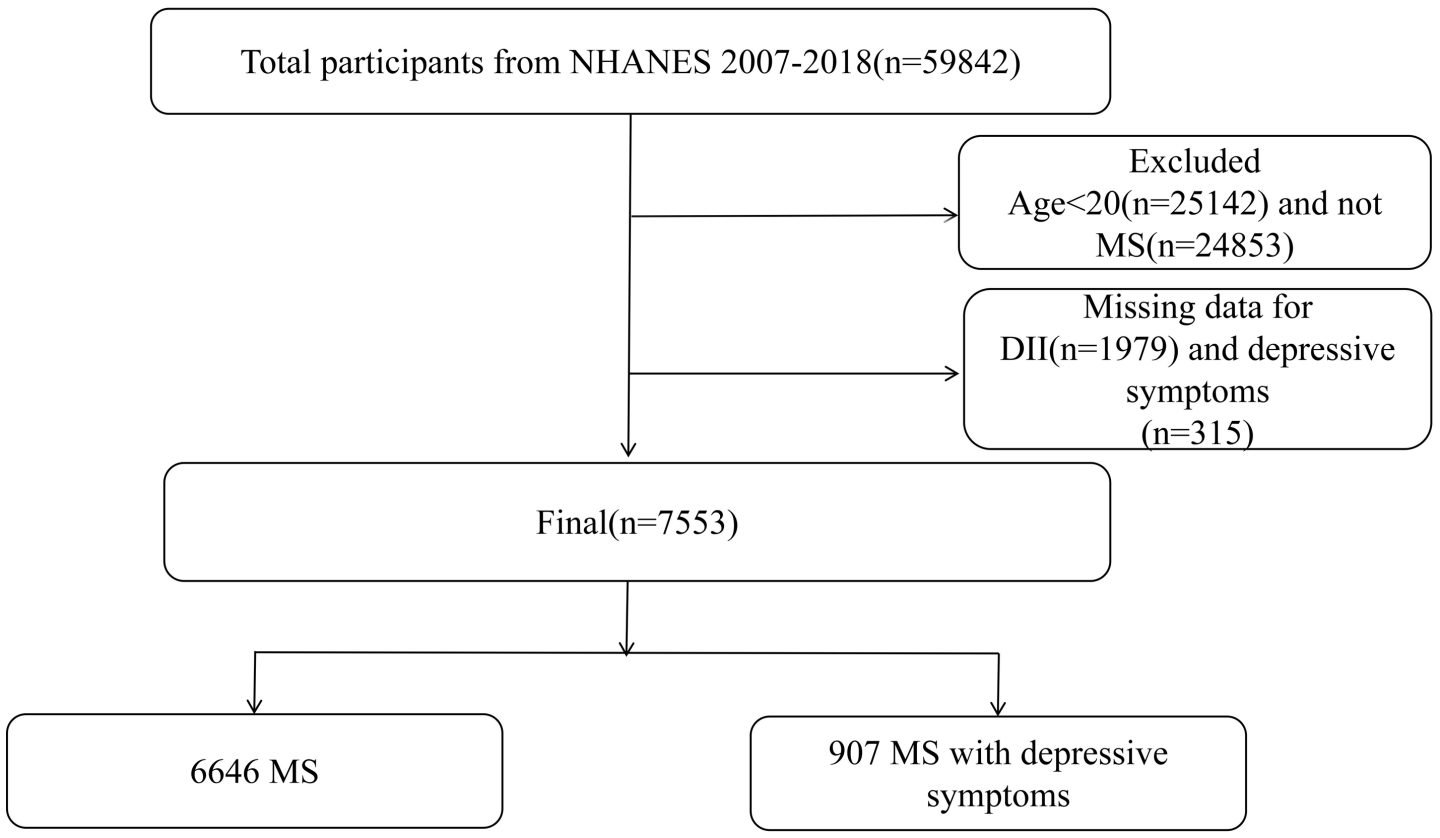
Flowchart of study participants. MS, metabolic syndrome; DII, dietary inflammatory index.

Our study utilized NHANES data from the pre-pandemic period (2007–2018). Initially, the cohort included 59,842 participants. This study specifically targeted respondents aged 20 and older with MS, excluding those who did not provide dietary information. Additionally, participants with missing data on the depressive symptoms Patient Questionnaire were also excluded, as they may not have been able to assess their depressive symptoms. A total of 7,553 participants were included in the complete case analysis. The sample selection process is detailed in Figure 1.

### 2.2 The definition of DII

The exposure variable in this study is the DII (16). The DII is a widely utilized parameter for assessing overall dietary inflammation, and its calculation method has been well-documented in the literature (17). The general procedure for calculating the DII for specific food components involves processing raw dietary data, calculating Z-scores, centering these Z-scores, and then multiplying them by the “overall inflammatory effect score.” The sum of the DII scores across all food components yields the final DII score. In this study, the DII was calculated using dietary interview data from the NHANES, specifically incorporating 28 out of 45 food components. Certain components, such as flavan-3-ols, flavonoids, flavonols, anthocyanins, pepper, thyme/oregano, and rosemary, were excluded due to the lack of data in the NHANES 2007–2018 dataset. Given that the consumption of many of these missing components was low within the population, their exclusion had minimal impact on the overall DII score. The final DII scores were derived from two 24-h dietary recall datasets to minimize error. The DII was calculated using the “dietaryindex” package.

### 2.3 The definition of depressive symptoms in people with MS

NHANES utilizes the validated Patient Health Questionnaire (PHQ-9) to assess depressive symptoms, with a reported sensitivity and specificity of 88%. The PHQ-9 is a reliable screening tool designed to evaluate participants’ depressive symptoms over the past two weeks, including indicators such as insomnia, reduced appetite, and feelings of loneliness. Each of the nine questions is rated on a four-point scale reflecting symptom frequency (0 = Not at all, 1 = Several days, 2 = More than half the days, 3 = Nearly every day). The total PHQ-9 score is the sum of all individual item scores. Participants with a total score of 10 or higher are classified as experiencing mild to severe depressive symptoms (18). This study will analyze the odds of depressive symptoms and use the total PHQ-9 score as the outcome variable.

MS is assessed based on five key criteria: waist circumference (WC), triglycerides (TG), high-density lipoprotein (HDL), blood pressure, and fasting glucose (FG). According to the ATP III guidelines, a diagnosis of MS is made when three or more of the following conditions are present:

Abdominal obesity (WC > 40 inches in men, > 35 inches in women);

TG ≥ 150 mg/dL;

HDL < 40 mg/dL in men or < 50 mg/dL in women;

Systolic blood pressure ≥ 130 mmHg or diastolic blood pressure ≥ 85 mmHg;

FG ≥ 110 mg/dL.

### 2.4 Covariates

Covariates in this study include age, gender, race, smoking status, family income-to-poverty ratio (PIR), abdominal obesity, hypertension, diabetes, educational level, and marital status. These covariates were derived from various sections of the NHANES database, including demographics, examination, diabetes questionnaires, and smoking questionnaires. A comprehensive description of all variables is available on the official NHANES website.^1^ All data used in this study were obtained from the NHANES website.^2^

### 2.5 Statistical analysis

Multivariable logistic regression models were used to examine the independent association between the DII and depressive symptoms in participants with MS. DII levels were converted from continuous to categorical variables (tertiles), and trend tests were conducted to assess the linear relationship between DII and the odds of depressive symptoms in this population. In Model 1, no adjustments for covariates were made. Model 2 adjusted for age, gender, race, and the PIR. Model 3 further adjusted for age, gender, race, PIR, smoking status, hypertension, diabetes, and abdominal obesity. Additionally, restricted cubic spline (RCS) regression analysis was performed to explore the potential non-linear relationship between DII and depressive symptoms in participants with MS. Subgroup analyses were conducted to investigate the association between DII and depressive symptoms in participants with MS in various subgroups, including age, gender, race, PIR, educational level, smoking status, marital status, diabetes, hypertension, and abdominal obesity. Data analysis was carried out using R software. A two-tailed *p*-value of <0.05 was considered statistically significant for all analyses.

## 3 Results

### 3.1 Baseline characteristics of participants

A total of 7,553 adults with MS participated in this study, including 907 depressive symptoms in participants with MS, as well as 6,646 participants with MS without depressive symptoms. Additionally, Table 1 presents the general demographic characteristics of the participants who were excluded and included in this study.

**Table 1:** General characteristics of participants in NHANES 2007–2018.

Characteristics	Overall	MS with depressive symptoms	MS	*p* value
	*n* = 7,553	*n* = 907	*n* = 6,646	
Gender				<0.001
Male	3,750 (49.6%)	324 (35.7%)	3,426 (51.5%)	
Female	3,803 (50.4%)	583 (64.3%)	3,220 (48.5%)	
Educational level				<0.001
High school or below	4,007 (53.1%)	568 (62.6%)	3,439 (51.7%)	
Above high school	3,546 (46.9%)	339 (37.4%)	3,207 (48.3%)	
Race				<0.001
Mexican American	1,187 (15.7%)	125 (13.8%)	1,062 (16.0%)	
Other Hispanic	820 (10.9%)	134 (14.8%)	686 (10.3%)	
Non-Hispanic White	3,233 (42.8%)	396 (43.7%)	2,837 (42.7%)	
Non-Hispanic Black	1,625 (21.5%)	192 (21.2%)	1,433 (21.6%)	
Other Race	688 (9.11%)	60 (6.62%)	628 (9.45%)	
Marital status				<0.001
Married/Living with partner	4,618 (61.1%)	446 (49.2%)	4,172 (62.8%)	
No or unclear	2,935 (38.9%)	461 (50.8%)	2,474 (37.2%)	
Smoking status				<0.001
Never or unclear	3,822 (50.6%)	356 (39.3%)	3,466 (52.2%)	
Former	2,233 (29.6%)	244 (26.9%)	1989 (29.9%)	
Current	1,498 (19.8%)	307 (33.8%)	1,191 (17.9%)	
PHQ	3.85 ± 4.87	14.5 ± 4.71	2.41 ± 2.56	0.000
Age, year				<0.001
20–40	1,318 (17.5%)	168 (18.5%)	1,150 (17.3%)	
41–60	2,761 (36.6%)	405 (44.7%)	2,356 (35.4%)	
>60	3,474 (46.0%)	334 (36.8%)	3,140 (47.2%)	
DII	1.37 ± 1.65	1.79 ± 1.54	1.32 ± 1.66	<0.001
PIR				<0.001
Poor	1,544 (20.4%)	322 (35.5%)	1,222 (18.4%)	
Not poor or unclear	6,009 (79.6%)	585 (64.5%)	5,424 (81.6%)	
Hypertension				0.257
Yes	6,542 (86.6%)	797 (87.9%)	5,745 (86.4%)	
No	1,011 (13.4%)	110 (12.1%)	901 (13.6%)	
WC				0.012
Yes	7,457 (98.7%)	887 (97.8%)	6,570 (98.9%)	
No	96 (1.3%)	20 (2.2%)	76 (1.1%)	
Diabetes				0.002
Yes	3,754 (49.7%)	495 (54.6%)	3,259 (49.0%)	
No	3,799 (50.3%)	412 (45.4%)	3,387 (51.0%)	

Mean (±SE) for continuous; n (%) for categorical. MS, metabolic syndrome; DII, dietary inflammatory index; PIR, ratio of family income to poverty; WC, Abdominal obesity; PHQ, patient health questionnaire.

### 3.2 Association between DII and depressive symptoms in participants with MS

Table 2 illustrates the comparison of DII components scores between the MS with depressive symptoms group and the MS group. Table 3 illustrates the comparison of DII components consumption between the MS with depressive symptoms group and the MS group. Table 4 presents the association between the DII and depressive symptoms in participants with MS. The DII was categorized into tertiles (T1: 0.25, T2: 1.57, T3: 2.64) and analyzed using logistic regression. Higher DII scores were associated with an increased likelihood of depressive symptoms in participants with MS compared to the T1 group. Specifically, for the T2 group, the odds ratio (OR) was 1.36 (95% CI: 1.13–1.63, *p* < 0.001), and for the T3 group, the OR was 1.98 (95% CI: 1.67–2.36, *p* < 0.001). After full adjustment for potential confounders, the odds of depressive symptoms remained significantly elevated in the higher DII tertiles compared to the T1 group (T2: OR = 1.13, 95% CI: 0.93–1.37, *p* < 0.001; T3: OR = 1.36, 95% CI: 1.13–1.65, *p* < 0.001).

**Table 2:** Comparison of DII components scores between the MS with depressive symptoms group and the MS group.

Variables	Overall	MS	MS with depressive symptoms	*p* value
	*n* = 7,553	*n* = 6,646	*n* = 907	
DII	1.37 (1.65)	1.32 (1.66)	1.79 (1.54)	<0.001
Alcohol	0.22 (0.14)	0.22 (0.14)	0.23 (0.13)	0.007
Vitamin B12	−0.03 (0.05)	−0.02 (0.05)	−0.03 (0.05)	0.004
Vitamin B6	−0.06 (0.18)	−0.07 (0.18)	−0.02 (0.19)	<0.001
β-Carotene	0.35 (0.28)	0.34 (0.28)	0.40 (0.24)	<0.001
Caffeine	0.08 (0.00)	0.08 (0.00)	0.08 (0.00)	0.001
Carbohydrate	−0.04 (0.06)	−0.04 (0.06)	−0.04 (0.06)	0.366
Cholesterol	−0.02 (0.08)	−0.02 (0.08)	−0.03 (0.08)	<0.001
Energy	−0.04 (0.12)	−0.04 (0.12)	−0.05 (0.12)	0.003
Total fatty acid	−0.02 (0.19)	−0.02 (0.19)	−0.04 (0.19)	<0.001
Dietary fiber	0.21 (0.40)	0.20 (0.41)	0.29 (0.38)	<0.001
Folate	0.12 (0.10)	0.11 (0.10)	0.12 (0.09)	0.136
Iron	0.00 (0.02)	0.00 (0.02)	0.00 (0.02)	<0.001
Magnesium	0.10 (0.22)	0.09 (0.22)	0.14 (0.21)	<0.001
MUFA	0.00 (0.01)	0.00 (0.01)	0.00 (0.01)	<0.001
Niacin	0.05 (0.12)	0.04 (0.12)	0.07 (0.11)	<0.001
n3 Polyunsaturated fatty acid	−0.11 (0.17)	−0.12 (0.17)	−0.08 (0.17)	<0.001
n6 Polyunsaturated fatty acid	−0.03 (0.08)	−0.03 (0.07)	−0.02 (0.08)	<0.001
Protein	0.00 (0.01)	0.00 (0.01)	−0.01 (0.01)	<0.001
PUFA	−0.02 (0.22)	−0.02 (0.22)	0.01 (0.22)	<0.001
Vitamin B2	0.00 (0.04)	0.00 (0.04)	0.00 (0.04)	0.006
Total saturated fatty acid	−0.12 (0.22)	−0.12 (0.22)	−0.14 (0.22)	0.046
Selenium	−0.09 (0.10)	−0.09 (0.10)	−0.06 (0.10)	<0.001
Vitamin B1	0.02 (0.05)	0.02 (0.05)	0.03 (0.05)	<0.001
Vitamin A	0.20 (0.16)	0.20 (0.16)	0.22 (0.15)	<0.001
Vitamin C	0.21 (0.23)	0.21 (0.23)	0.24 (0.21)	<0.001
Vitamin E	0.17 (0.27)	0.16 (0.27)	0.20 (0.25)	<0.001
Zinc	0.02 (0.21)	0.02 (0.21)	0.06 (0.20)	<0.001
Vitamin D	0.22 (0.24)	0.21 (0.24)	0.22 (0.24)	0.228

Data are presented as the mean and standard deviation. DII, dietary inflammatory index; MUFA, monounsaturated fatty acids; PUFA, polyunsaturated fatty acids.

**Table 3:** Comparison of DII components consumption between the MS with depressive symptoms group and the MS group.

Variables	Overall	MS	MS with depressive symptoms	*P* value
	n = 7,553	n = 6,646	n = 907	
Alcohol (g/d)	5.28 (17.5)	5.36 (17.3)	4.68 (18.7)	0.295
Vitamin B12 (mcg/d)	4.81 (5.30)	4.82 (5.13)	4.75 (6.43)	0.728
Vitamin B6 (mg/d)	1.92 (1.22)	1.95 (1.23)	1.75 (1.13)	<0.001
β-Carotene (mcg/d)	2,110 (3226)	2,163 (3078)	1718 (4132)	0.002
Caffeine (g/d)	0.15 (0.17)	0.14 (0.17)	0.17 (0.21)	0.001
Carbohydrate (g/d)	234 (101)	235 (101)	231 (101)	0.374
Cholesterol (mg/d)	289 (192)	293 (193)	260 (178)	<0.001
Energy (kcal/d)	1923 (789)	1933 (792)	1847 (765)	0.001
Total fatty acid (g/d)	74.2 (37.2)	74.8 (37.3)	70.2 (36.4)	0.001
Dietary fiber (g/d)	16.2 (8.65)	16.5 (8.74)	14.2 (7.74)	<0.001
Folate (mcg/d)	374 (202)	378 (202)	345 (192)	<0.001
Iron (mg/d)	14.1 (7.26)	14.2 (7.29)	13.2 (6.96)	<0.001
Magnesium (mg/d)	281 (141)	284 (141)	259 (139)	<0.001
MUFA (g/d)	26.5 (14.0)	26.7 (14.0)	24.9 (13.6)	<0.001
Niacin (mg/d)	23.4 (11.7)	23.6 (11.7)	21.8 (11.5)	<0.001
n3 Polyunsaturated fatty acid (g/d)	1.71 (1.06)	1.73 (1.07)	1.55 (0.99)	<0.001
n6 Polyunsaturated fatty acid (g/d)	15.1 (8.64)	15.2 (8.66)	14.2 (8.41)	<0.001
Vitamin B2 (mg/d)	1.94 (0.99)	1.95 (0.98)	1.90 (1.04)	0.177
Protein (g/d)	76.6 (33.5)	77.5 (33.7)	69.9 (31.5)	<0.001
PUFA (g/d)	17.0 (9.57)	17.1 (9.60)	15.9 (9.32)	<0.001
Total saturated fatty acid (g/d)	24.0 (13.1)	24.1 (13.1)	23.1 (12.9)	0.024
Selenium (mcg/d)	108 (51.2)	109 (51.5)	97.7 (48.2)	<0.001
Vitamin B1 (mg/d)	1.52 (0.73)	1.53 (0.73)	1.42 (0.69)	<0.001
Vitamin A (mcg/d)	603 (553)	606 (526)	575 (721)	0.212
Vitamin C (mg/d)	76.9 (69.5)	78.1 (69.3)	68.1 (70.7)	<0.001
Vitamin E (mg/d)	7.55 (4.88)	7.64 (4.92)	6.90 (4.51)	<0.001
Zinc (mg/d)	10.6 (6.67)	10.7 (6.72)	9.87 (6.18)	<0.001
Vitamin D (mcg/d)	4.46 (4.28)	4.50 (4.34)	4.16 (3.83)	0.014

Data are presented as the mean and standard deviation. DII, dietary inflammatory index; MUFA, monounsaturated fatty acids; PUFA, polyunsaturated fatty acids.

**Table 4:** Logistic regression on the association between DII and depressive symptoms in participants with MS.

	Model 1	*p* value	*p* for trend	Model 2	*p* value	*p* for trend	Model 3	*p* value	*p* for trend
	OR[95%CL]			OR[95%CL]			OR[95%CL]		
Continuous DII	1.20 [1.15, 1.26]	<0.001	-	1.13 [1.08, 1.19]	<0.001	-	1.09 [1.04, 1.14]	<0.001	-
DII-T1	Ref	-	<0.001	Ref	-	<0.001	Ref	-	0.001
DII-T2	1.36 [1.13, 1.63]	0.001		1.20 [0.99, 1.45]	0.059		1.13 [0.93, 1.37]	0.211	
DII-T3	1.98 [1.67, 2.36]	<0.001		1.57 [1.31, 1.88]	<0.001		1.36 [1.13, 1.65]	0.001	

Data are presented as OR [95% confidence interval]. OR, odds ratio; CI, confidence interval; PIR, ratio of family income to poverty. Model 1: no covariates were adjusted. Model 2: age, gender, race, and PIR were adjusted. Model 3: age, gender, race, PIR, educational level, smoking status, marital status, diabetes, hypertension, and abdominal obesity were adjusted.

### 3.3 Restricted cubic spline regression analysis

**Figure 2:**
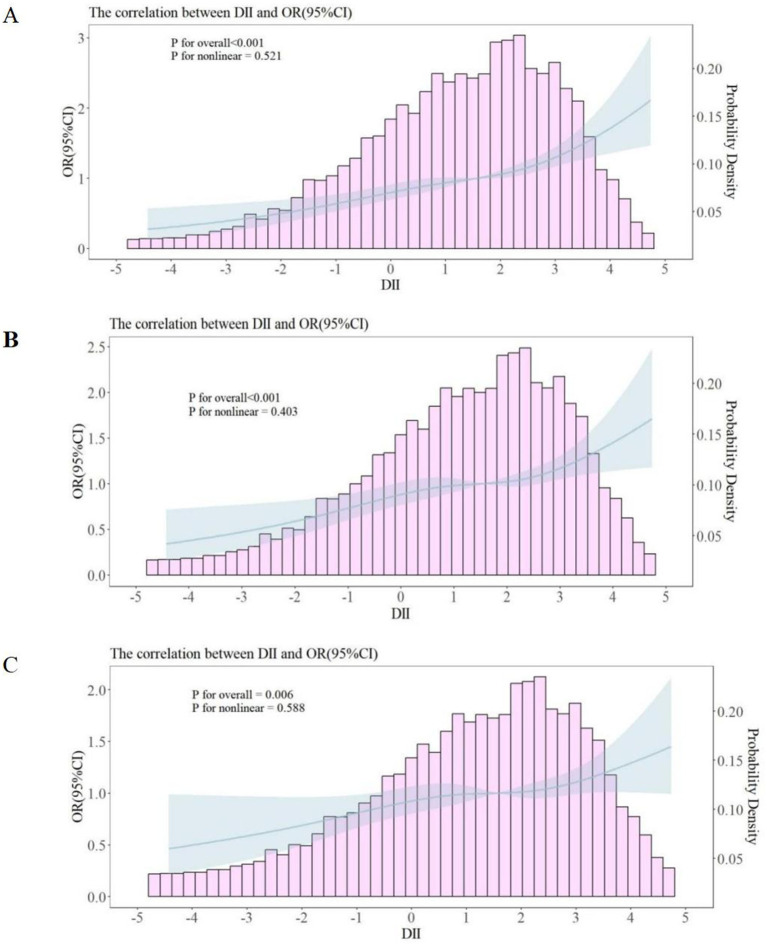
Restricted cubic spline curves between DII and depressive symptoms in participants with MS. OR, odds ratio; CI, confidence interval; PIR, ratio of family income to poverty. **(A)** No covariates were adjusted. **(B)** Age, gender, race, and PIR were adjusted. **(C)** Age, gender, race, PIR, educational level, smoking status, marital status, diabetes, hypertension, and abdominal obesity were adjusted.

**Figure 3:**
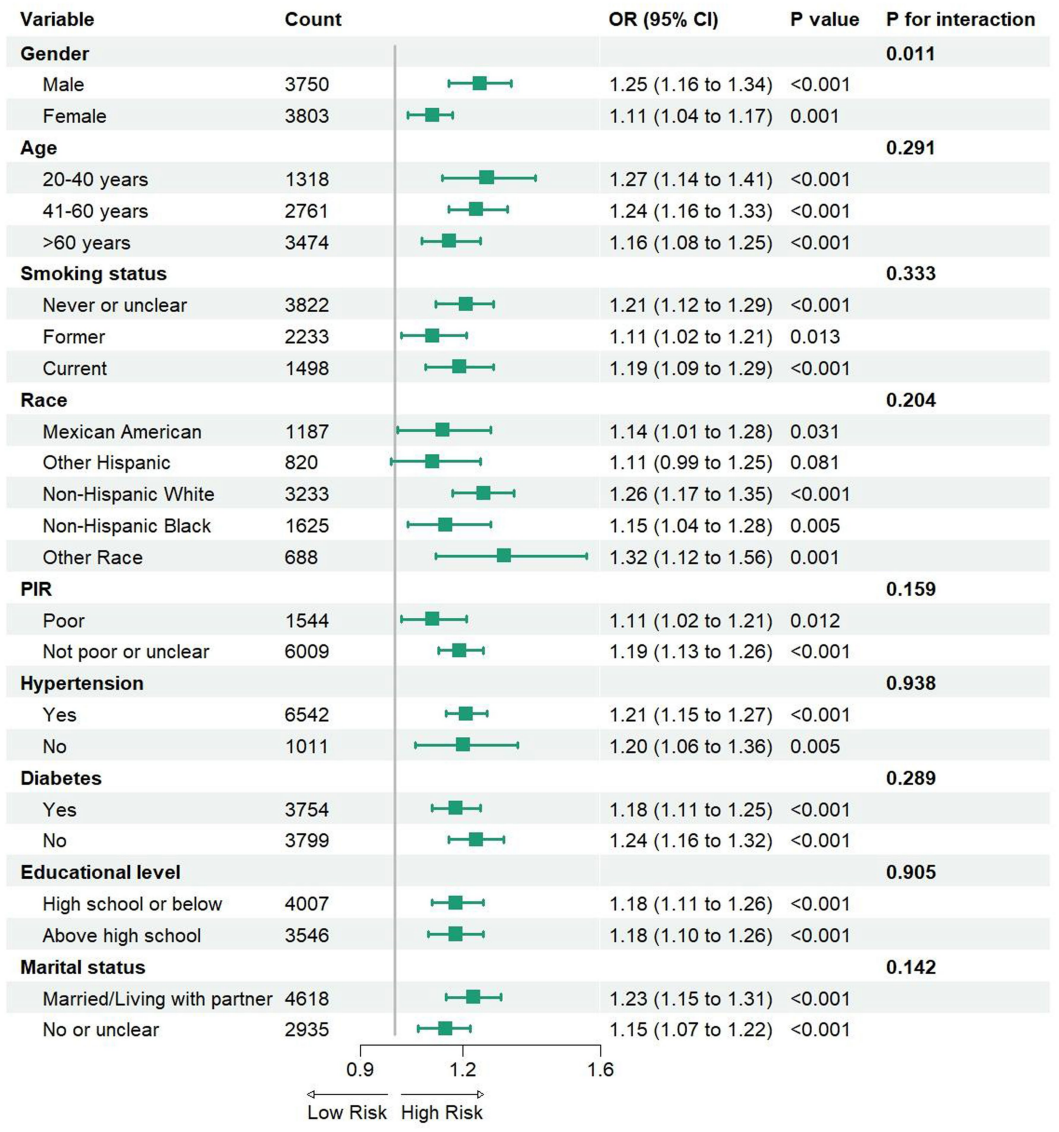
The Subgroup analysis between DII and depressive symptoms in participants with MS. PIR, ratio of family income to poverty; OR, odds ratio; CI, confidence interval.

In participants with MS, a RCS analysis revealed a nonlinear association between the DII and depressive symptoms in participants with MS. The models are presented sequentially as Model 1 (Figure 2A), Model 2 (Figure 2B), and Model 3 (Figure 2C). The analysis showed a positive correlation between DII scores and depressive symptoms in participants with MS, indicating that higher DII scores were associated with an increased likelihood of depressive symptoms in participants with MS (Figure 3).

### 3.4 Subgroup analysis

In this study, we conducted subgroup analyses and interaction tests to investigate the consistency of the relationship between the DII and depressive symptoms in participants with MS, stratifying participants by gender, age, smoking status, race, poverty level, hypertension, diabetes, abdominal obesity, educational level, and marital status (Figure 3). The results indicated that this association was not significantly correlated with different age groups, smoking status, race, PIR, diabetes, abdominal obesity, educational level, or marital status (interaction *p* > 0.05). However, a notable difference was observed in the subgroup analysis based on gender. A significant positive correlation was found in the male group (OR = 1.25, 95% CI: 1.16–1.34), which was markedly higher than that in the female group (OR = 1.11, 95% CI: 1.04–1.17). Furthermore, our findings suggest that the positive correlation between the DII and depressive symptoms in participants with MS remains consistent across different age groups, smoking statuses, races, PIR, diabetes, hypertension, abdominal obesity, educational levels, and marital statuses, indicating its potential applicability across various population contexts.

## 4 Discussion

In this cross-sectional study, we utilized a representative national sample from the United States. Our findings indicate a positive correlation between the DII and depressive symptoms in participants with MS, which persists even after adjusting for covariates. This association remains consistent across various subgroups, including age, smoking status, race, PIR, diabetes, hypertension, abdominal obesity, educational level, and marital status. RCS analysis further illustrates the nonlinear relationship between the DII and depressive symptoms in participants with MS. This correlation remained significant both before and after adjustment for covariates. The RCS curve suggests that higher DII levels are associated with an increased likelihood of depressive symptoms, implying that dietary-induced inflammation may serve as a potential predictor for the onset of depressive symptoms in participants with MS.

Multiple studies have highlighted a significant association between MS and depressive symptoms, contributing to a substantial economic burden (19). Existing research suggests that inflammation serves as a primary mechanism for the comorbidity of MS and depressive symptoms, particularly in relation to the activation of inflammatory factors such as IL-6 (20). In the context of obesity, the increase in white adipose tissue leads to a state of low-grade inflammation. This inflammatory state is a consequence of the accumulation of various inflammatory cells, including macrophages, CD8+ T cells, IL-17 producing CD4+ T cells, and natural killer cells (21). Furthermore, levels of adiponectin (an anti-inflammatory mediator) are decreased in obesity and increased with caloric restriction (22).

In research on MS, various dietary patterns have been investigated, including the Mediterranean diet, plant-based diets, and anti-inflammatory diets. Key components of these dietary patterns significantly influence processes such as glucose metabolism and insulin sensitivity, which are closely linked to the development and progression of MS. Furthermore, many neurotransmitters that regulate mood and behavior are synthesized from dietary precursors, making adequate intake of proteins, vitamins, and minerals essential for the production and function of these neurotransmitters (23). Higher inflammatory dietary patterns have been associated with an increased risk of type 2 diabetes, obesity, MS, depressive symptoms, and certain cancers, with this phenomenon being more pronounced in older adults (24). Cross-sectional studies have shown that a legume-nut dietary pattern is linked to an increased risk of MS, particularly among individuals with irregular physical activity (25). In contrast, a “vegetarian” dietary pattern has been shown to improve depressive symptom scores, psychosocial functioning, quality of life, body mass index, cholesterol levels, low-density lipoprotein, and diastolic blood pressure in participants with depressive disorders related to MS (26).

As far as we know, no previous studies have explored the relationship between the DII and depressive symptoms in participants with MS. To address this research gap, our study aimed to investigate the association between depressive symptoms in participants with MS and DII. The results suggest that higher DII scores are positively correlated with an increased likelihood of depressive symptoms in these participants. Subgroup analysis revealed no significant differences among Hispanic groups, which may be related to variations in dietary patterns. Previous studies have indicated that traditional Hispanics consume more fruits than non-Hispanic Blacks and non-Hispanic Whites (27). Since fruits are rich in anti-inflammatory compounds, this dietary habit may help mitigate the risk of depressive symptoms in participants with MS. Interestingly, the impact of the DII on depressive symptoms was more pronounced in men, which contrasts with earlier studies suggesting that women are more likely to experience depressive symptoms associated with MS (28). While our study also found a higher odds of depressive symptoms in females, this may be due to the predominance of men in the study population used for DII assessment. Research has shown that women often cope more effectively with lifestyle disruptions and tend to make more restrictive food choices, whereas men are more likely to follow dietary patterns associated with inflammation (29). Furthermore, studies have suggested that emotional disorders such as depressive symptoms and anxiety are significant risk factors for the development of MS in men, while in women, age and marital status are often more prominent risk factors (30). The primary focus of this study is MS, and given that men tend to exhibit more emotional disorders, gender differences in the prevalence of depressive symptoms are noteworthy. Several studies have shown that male gender is more strongly associated with depressive symptoms in individuals with MS, reinforcing the findings from our study (31, 32).

However, further experimental research is necessary to validate this hypothesis. Our study has several limitations. First, due to the inherent constraints of cross-sectional study design, we are unable to establish a causal relationship between the DII and depressive symptoms in individuals with MS. Therefore, large-scale prospective studies are needed to better understand the temporal relationship between these variables. Second, dietary data were based on self-reported recall from questionnaires, which may introduce potential biases. Although we averaged data from two separate sessions to minimize error, reliance on self-reported dietary intake inevitably introduces recall bias. Additionally, missing dietary data may lead to an underestimation of the DII score. Finally, since our study sample was limited to U.S. adults, the generalizability of our findings to other populations may be limited.

## 5 Conclusion

This study identified a positive correlation between higher consumption of pro-inflammatory diets and an increased likelihood of depressive symptoms in individuals with MS. These findings suggest that dietary interventions designed to reduce the DII may help mitigate inflammation and lower the odds of depressive symptoms. However, MS with comorbid depressive symptoms is a multifaceted condition influenced by numerous factors. Therefore, a comprehensive and personalized approach to treatment is necessary to address the underlying complexities of this comorbidity.

## Data Availability

Publicly available datasets were analyzed in this study. This data can be found at: https://www.cdc.gov/nchs/nhanes/.

## Footnotes

^1^ https://www.cdc.gov/nchs/nhanes

^2^ https://wwwn.cdc.gov/nchs/nhanes/
